# Levonorgestrel-Releasing Intrauterine System as a Contraceptive Method in Nulliparous Women: A Systematic Review

**DOI:** 10.3390/jcm9072101

**Published:** 2020-07-03

**Authors:** Magdalena Zgliczynska, Karol Kocaj, Iwona Szymusik, Magdalena Maria Dutsch-Wicherek, Michal Ciebiera, Katarzyna Kosinska-Kaczynska

**Affiliations:** 1 2nd Department of Obstetrics and Gynecology, Centre of Postgraduate Medical Education, 01-809 Warsaw, Poland; zgliczynska.magda@gmail.com (M.Z.); katarzyna.kosinska-kaczynska@cmkp.edu.pl (K.K.-K.); 2 Medical University of Silesia, 40-055 Katowice, Poland; kocajkarol.kk@gmail.com; 3 1st Department of Obstetrics and Gynecology, Medical University of Warsaw, 02-015 Warsaw, Poland; iwona.szymusik@gmail.com; 4 Department of Psychiatry, Centre of Postgraduate Medical Education, 01-809 Warsaw, Poland; dutsch.wicherek@gmail.com

**Keywords:** contraception, intrauterine device, intrauterine system, levonorgestrel-releasing intrauterine system, nulliparous, nulliparity

## Abstract

The aim of this review was to summarize the available evidence about the use of levonorgestrel-releasing intrauterine system (LNG-IUS) as a contraceptive method in nulliparous women. For this purpose, studies evaluating the efficacy, safety, bleeding pattern, satisfaction and discontinuation of the levonorgestrel-releasing intrauterine system in nulliparous women were analyzed. Only original research articles published in English between 1990–27th March 2020 were considered eligible. Reviews, book chapters, case studies, conference papers, opinions, editorials and letters were excluded. The systematic literature search of PubMed/MEDLINE, Scopus, Embase and Cochrane Library databases identified 816 articles, 23 of which were analyzed. The available evidence indicates that LNG-IUS is an effective and safe contraceptive method for nulliparous women that achieves high levels of satisfaction among patients. Moreover, nulliparous women seem to experience fewer expulsions than parous ones. Bleeding pattern is acceptable for the majority of patients, and bleeding disorders mainly occur in the first months after the insertion. More in-depth, long-term prospective studies are needed in this patient group to determine risk factors for the occurrence of side effects and associated discontinuations, which should not, however, delay the wider use of the method in this group, given the number of advantages.

## 1. Introduction

Contraception (or birth control) is a deliberate use of methods or devices to prevent pregnancy as a consequence of sexual intercourse. Birth control has been used since ancient times, but its efficacy and safety has always been a problematic matter. A great progress in contraception research was observed in the 20th century, when contraceptives improved women′s quality of life (QoL) and reduced various health conditions related to unplanned pregnancies [1,2]. Due to this revolution, couples may currently have sexual intercourse at any desired time [3].

Intrauterine devices are a form of long-acting reversible contraception (LARC) methods. They are placed in a woman′s uterus. The new era of hormonal intrauterine devices (IUD) started when Luukkainen (1976) replaced the IUD copper filament with a small reservoir releasing constant daily doses of levonorgestrel [4]. Most of its contraceptive effect results from the hormonally-induced endometrial atrophy, and from the physicochemical changes of the cervical mucus [4]. Nowadays, hormonal IUDs are believed to result in one of the greatest satisfaction among users [5,6]. According to Canadian Contraception Consensus, LARCs are the most effective reversible contraceptive methods and have the highest continuation rates [7,8,9]. The numbers are satisfying—0.2% of women (both multiparous and nulliparous) using hormonal IUDs experience an unintended pregnancy within the first year of using this method [10]. Beyond its high efficacy, it offers a variety of potential non-contraceptive therapeutic benefits, e.g., it contributes to partial or complete menstrual suppression, improvement in dysmenorrhea and amelioration in pain associated with endometriosis and adenomyosis [11]. In addition to the aforementioned short-term effects, it also reduces the lifetime risk of endometrial cancer and ovarian cancer [12].

Most gynecological societies support the use of hormonal IUDs, e.g., the American College of Obstetricians and Gynecologists (ACOG) has stated in their guidelines that LARCs are a safe and acceptable means of LARC for both adults and adolescents, as well as parous and nulliparous women [13]. In most situations the advantages seem to outweigh the potential risks. According to available data, the use of LARCs in the United States (including hormonal IUDs) increased almost three times from 2009 (2.1%) to 2012 (5.9%) [14]. As stated by Lohr et al., IUDs should be routinely included in the contraception options offered to the majority of women, including those who did not give birth [15]. Even with many positive expert opinions and studies conducted in large groups, hormonal IUDs still trigger a lot of skepticism [16]. As found in study by Madden et al., only 31% of contraception providers consider an IUD appropriate for adolescents and young women [17]. In a different study, Luchowski et al. found that only two thirds of gynecologists considered IUDs appropriate for nulliparous women [18]. Moreover, numerous young women seem to have limited awareness and information about IUDs [19,20]. According to the Contraceptive CHOICE Project and other similar studies, more than half of adolescent patients preferred LARC methods over non-LARC ones after being properly advised and taught about contraceptive methods and their potential advantages and disadvantages [20,21]. Therefore, unlimited access to reliable sources of information is crucial.

To this day, several systematic reviews on the use of IUD in adolescents and nulliparous women have been conducted. Among those published in the recent years, the main topic became the IUD insertion procedure and pain management in this specific population [22,23,24]. They indicated higher rates of difficulties in insertion, insertion failure and pain during insertion in nulliparous, but at the same time, highlight the existence of potentially helpful interventions, such as cervix preparation with the use of misoprostol or lidocaine anesthesia [22,23,24]. Other available summaries demonstrated high level of safety and encouraging continuation rates of IUDs in young women [25,26,27]. However, as several new important articles on this topic have been published in the recent past, this review aims to provide a broad look at levonorgestrel-releasing intrauterine system (LNG-IUS) outcomes in the population of nulliparous women, regardless of their age.

In summary, as concerns about the use of LNG-IUS in nulliparous women may still limit the its availability in a large group of patients, an up-to-date overview on the available evidence is needed. Therefore, the objective of this systematic review was to summarize and critically appraise the available evidence about the use of LNG-IUS as a contraceptive method in nulliparous women.

## 2. Material and Methods

The protocol for this systematic review was registered at PROSPERO—an International Prospective Register of Systematic Reviews (CRD42019139169), and may be accessed at https://www.crd.york.ac.uk/prospero/display_record.php?RecordID=139169.

The inclusion and exclusion criteria are summarized in Table 1.

Papers regarding the insertion procedure itself or pain perception and management were not considered, since major systematic reviews on the topics have been published by other authors in recent years [22,23,24].

The following LNG-IUS outcomes were taken into account: efficacy (the rate of unintended pregnancies and Pearl Index (PI)), safety (the rate and kind of side effects), bleeding pattern, satisfaction (various satisfaction rates) and continuation (the rate of continuation and reasons for discontinuation). PI was defined as the number of pregnancies that occurred divided by the number of treatment cycles multiplied by 1300 times [28].

Four electronic databases were searched: MEDLINE (through PubMed), Scopus, Embase and CENTRAL. The search strategy for each database are presented in Table S1. The last search was run on 27th March 2020 on each database. We also reviewed the reference lists of selected studies, however, no additional records meeting the inclusion criteria were noted. It was not necessary to contact the authors of retrieved research articles for additional information.

A systematic literature search retrieved 816 studies. Duplicates were removed using the automatic EndNote X9 (Clarivate Analytics, Philadelphia, Pennsylvania, United States) duplicate finder, followed by a manual search. Titles and abstracts of remaining articles were independently screened by three study authors. Full-text versions of studies potentially eligible were assessed by three other authors. Details regarding the selection process are summarized in a custom-built PRISMA flow chart in Figure S1. In the next step, authors collected the baseline characteristics of the participants and outcomes for each selected study using a self-developed data extraction sheet. Any disagreements were resolved through discussion and consensus.

The risk of bias for the interpretation of the data on the use of LNG-IUS in nulliparous in the selected studies was analyzed with the use the Newcastle-Ottawa Quality Assessment Scale, modified by the authors for the needs of this review [29] (Table S2). A study was awarded one star each star-rated feature within the Selection and Outcome categories. A maximum of two stars could be awarded for Comparability. Finally, a study was rated as having a low risk of bias if it gained: 3 or 4 stars in “Selection” AND 1 or 2 stars in “Comparability” AND 2 or 3 stars in “Outcome”; moderate risk of bias if: 2 stars in “Selection” AND 1 or 2 stars in “Comparability” AND 2 or 3 stars in “Outcome”; high risk of bias if: 0 or 1 star in “Selection” OR 0 stars in “Comparability” OR 0 or 1 stars in “Outcome”.

## 3. Results

As a result of the described search procedure, 23 articles that meet all the inclusion criteria were retrieved [30,31,32,33,34,35,36,37,38,39,40,41,42,43,44,45,46,47,48,49,50,51,52]. Basic data on the research works included in this systematic review are collected in Table 2.

In only one of the studies that met the previously assumed criteria, the study group consisted of patients using low-dose IUS [35]. Both LNG-IUS 8 µg/24 h and LNG-IUS 13 µg/24 h were proved by Gemzell-Danielsson et al. to be comparably highly effective, safe and gained with similar high satisfaction and continuation rates among users [35]. Nevertheless, it needs to be underlined that there are other studies on low-dose LNG-IUS usage in a mixed population-both parous and nulliparous women, in which their general safety and effectiveness were demonstrated, however, no separate calculations on this review outcomes of interest depending on the parity were reported [54,55,56]. The vast majority of available studies comparing the use of different types of IUS in a group of nulliparous women focus on the procedure of insertion and accompanying pain, which was the subject of other systematic reviews [22,23,24].

## 4. Discussion

### 4.1. Efficacy

A total of 12 out of 23 articles reported data on the pregnancy rates in groups of nulliparous LNG-IUS users [31,32,34,35,36,39,43,45,46,49,50,51]. Considering only those research papers in which the observation period was strictly defined, 11 pregnancies occurred in 9887 women-years of observation (based on eight studies: [31,32,35,36,45,46,49,51]). This amounts to a PI of about 0.11. However, it should be noted that it is uncertain whether women included in various studies used other methods of contraception simultaneously (e.g., barrier), or whether there were any other factors potentially distorting this indicator. The reported pregnancies were mostly ectopic—five out of seven in the study conducted by Gemzell-Danielsson et al. [35]. As regards the study by Teal et al. ectopic pregnancies accounted for 67% of all pregnancies in the study group (nulliparous and parous women analyzed jointly) [46].

### 4.2. Expulsion

A total of 14 out of 23 articles investigated the occurrence of expulsion in the analyzed population [31,32,34,35,36,37,38,39,41,43,45,46,49,51]. Using analogous calculations, 144 expulsions occurred during 11365 women-years (0.01 expulsion in 1 women-year; based on: [31,32,35,36,37,38,41,45,46,49,51]). Moreover, among six studies that compared expulsion indices depending on parity, five reported significantly lower numbers of expulsion in nulliparous than in the parous women [34,35,38,41,46], whereas Bahamondes et al. did not find a significant difference between groups [32]. Moreover, Madden et al. reported a decreased risk of LNG-IUS expulsion in nulliparous women (hazard ratio 0.59 (95% CI 0.44–0.78)). Interestingly, the authors also observed a doubled expulsion risk in females aged 14–19 compared to older women, but such a relationship is hard to explain [38]. However, the risk of expulsions remains low, and most cases may be recognized almost immediately, yet only unrecognized expulsions are considered clinically important [57].

### 4.3. Continuation

A total of 15 out of 23 articles reported data on the continuation rates in the studied population [30,31,32,35,36,37,39,41,43,45,47,48,49,51,52] (Table 3). In the studies assessing the rate of continuing treatment after the first year, continuation rates ranged from 73% (Kaislasuo et al.) up to 93% (Hall et al.). However, Kaislasuo et al. also considered 16% of lost to follow-up cases as a discontinuation [36,37]. For longer follow-ups, the rates were as follows: 3 years—50–58% [35,47]; 5 years—11–54% [47,49]. Apart from expulsion, the most common reasons for discontinuation in the retrieved studies were: bleeding, pain, will to conceive. Hall et al. reported that nulliparas using LNG-IUS had significantly higher continuation rates compared to those using Copper IUS (Cu-IUS) [36]. Similarly, Abraham et al. found that nulliparous women were more likely to discontinue Cu-IUS and implants than parous, but it was not reported for the group consisting of LNG-IUS users [30]. This finding was also confirmed by other authors [32,49,52]. In a study conducted by Teunissen et al., continuation rates in women with parity ≥ 2 were noticeably higher than in nulliparous ones at all follow-up visits (first year—77% vs. 81%; second year—64% vs. 72%; third year—50% vs. 65%; fourth year—38% vs. 59%; fifth year—11% vs. 24%; authors did not specify the level of statistical significance for those differences) [47]. Similar results were obtained on the basis of a crossed-design synthesis of randomized controlled trials (RCT) and observational study performed by Vaitsakhovich et al.. The continuation rates for parous women were 90% and 81% after 1 and 2 years, and the respective values for nulliparas accounted for 73% and 68% [48]. Gemzell-Danielsson et al. found that, after the first year of use, the continuation rate was significantly higher in parous women. However, after 3 years, there were no differences according to parity [35]. An interesting relationship was also found by Kaislasuo et al., who reported a significant association between an increasing fundal uterine width and the risk of discontinuation due to pain [37].

It is worth noting in this section that the number of patients continuing a contraceptive method to some extent reflects the degree of patient satisfaction and adjustment of the method to her needs. It may also provide information about her cooperation with the doctor [58]. Therefore, studies on nulliparous women involving in-depth analyses of discontinuation reasons—with and without the exclusion of patients who discontinued LNG-IUS only for will to conceive—might provide valuable information on this matter.

### 4.4. Bleeding Pattern

Bleeding profile disturbances are a rare yet probable scenario during LNG-IUS use, including among nulliparous women. The unacceptable bleeding pattern was one of the most common reason for LNG-IUS discontinuation, but still, such a situation occurred within a very small percentage of nulliparous users—from 2% during the first year of use, up to 6% during the fifth year of usage [31,32,49,51]. Nevertheless, bleeding pattern seems to be a crucial aspect of contraception for women, since Marions et al. reported that the level of satisfaction was dependent on the frequency and volume of bleeding and dysmenorrhea [39]. Several authors obtained similar amenorrhea rates in nulliparous LNG-IUS users—from 18% to 26% at the end of the first year of use, and even up to 36% at the end of the third year in the study by Schreiber et al. [33,35,36,37,39,40,44,45]. Moreover, the latter found that: infrequent bleeding rate increased from 12% to 30%, frequent bleeding rate decreased from 29% to 4%, prolonged bleeding rate decreased from 50% to 2% and irregular bleeding rate decreased from 39% to 4% during the last 90 days of the second year, in comparison to the first 90 days of usage. The bleeding patterns did not vary between parous and nulliparous women. Darney et al. also found no significant differences in the percentage of amenorrhea between parities [33]. Other authors also reported a decrease in the percentage of dysmenorrhea, spotting and irregular bleeding over time [39,45]. Suhonen et al., who aimed to compare LNG-IUS and oral contraceptives (OCs) in young nulliparous women, found that the use of LNG-IUS resulted in a more effective alleviation of dysmenorrhea, and a decreased amount of blood loss and bleeding days, than with OCs [45]. Kaislasuo et al. reported a relation between uterine cavity size and the bleeding pattern—decreasing uterine cavity size correlated with fewer spotting days and fewer days of pain. Moreover, baseline scanty menstrual bleeding predicted amenorrhea at 10–12 months (OR 8.17 (95% CI 1.38–48.21)), and so did smoking (OR 8.23 (95% CI 1.76–38.56)) [37]. Gemzell-Danielsson et al. studied the satisfaction of nulliparous users with bleeding pattern during the use of LNG-IUS—up to 73% of eight LNG-IUS users and 71% of 13 LNG-IUS users stated that they were ‘very satisfied’ or ‘somewhat satisfied’ with their menstrual bleeding pattern [35].

### 4.5. Other Side Effects

Among the retrieved articles, only one partial perforation, a case of myometrial embedment, was reported by the authors of the studies [35]. Moreover, Hall et al. reported one case of endometritis [36]. Apart from Gemzell-Danielsson et al., who calculated the crude rate of pelvic inflammatory disease (PID) over up to 3 years at 1% [35], other authors did not report this or any other serious side effects. A broader analysis of mild side effects was carried out by Suhonen et al., who compared the occurrence of selected symptoms at baseline and after 1 year of LNG-IUS use. They reported an increase in abdominal/back pain (34% vs. 55%), headache (56% vs. 60%), acne (39% vs. 59%), breast tenderness (33% vs. 37%), mood swings (52% vs. 57%), a stable percentage of depressive mood (45% for both assessment periods) and a decrease in irritability (58% vs. 52%). In contrast to subjectively experienced edema and weight gain in the LNG IUS group, no statistically significant increase in weight was noted [45].

There is still relatively little data in the available literature regarding the long-term safety of IUD use, especially regarding fertility. Nevertheless, those available do not indicate an increased risk of infertility [59,60].

### 4.6. Satisfaction

Only five out of 23 studies reported on the satisfaction with contraception method in nulliparous LNG-IUS users, and the rates ranged from 76% to 96% [35,39,42,45,52], with most studies reporting satisfaction in above 90% of participants (data summarized in Table 4).

It is worth mentioning that Suhonen et al. reported that significantly more LNG-IUS users wanted to continue the method after the study than in case of OC group (88% vs. 68%) [45]. Marions et al. also noted that the satisfaction rate was better in the youngest age group (≤ 20 years) than in the oldest studied group (≥ 31 years; 75% and 59%, respectively) [39].

### 4.7. Limitations and Strengths

The greatest advantage of this review is the fact that it contains the most up-to-date and extensive summary of the available data on the use of LNG-IUS in nulliparous women. Conversely, one of the biggest limitations is the paucity of quantitative analyses. This is due to the fact that the individual studies mostly used different methods to assess the various aspects of LNG-IUS use. Another noteworthy aspect is the problem with the nomenclature and different inclusion criteria of nulliparous or nulligravidas women in analyzed studies. Very few studies have defined this population in detail, e.g., by reporting the percentage of women who have undergone early pregnancy termination, which may be the source of some bias. Regrettably, not all reported basic outcomes, e.g., about the occurrence of unwanted pregnancies or perforations. In addition, the studies differed from basic characteristics of the population to the type of LNG-IUS applied. The vast majority of them investigated Mirena or other comparable in levonorgestrel dose and size IUS, which did not allow us to analyze the impact of these factors on outcomes of interest. What is more, in the generally available literature, there are still very few studies directly comparing different types of IUDs, which might be particularly important from a clinical point of view [54]. Nevertheless, some researchers attempt to analyze the purposefulness of adjusting the type of LNG-IUS to individual patients. For example, Wildemeersch et al.—who examined the uterine cavity size in over 400 nulliparous women seeking IUD insertion, and came to the conclusion that the vast majority of them have too narrow a uterine cavity to fit conventional IUD (32 mm)—suggested that smaller inserts might potentially be more appropriate in this group [61]. The risk of bias analysis indicated that, in terms of data on the usage of LNG-IUS in nulliparous women, most of the studies included in the review showed low bias risk (*N* = 12, 52%). Nevertheless, the remaining 11 studies was characterized by moderate or high bias risk (*N* = 2 (9%) and *N* = 9 (39%) respectively). Most of them rated low in the Comparability section, mainly due to the fact that nulliparous population has not been minutely characterized, which may potentially be the source of some bias. For details of the assessment see Table S2.

## 5. Conclusions

The review indicates that LNG-IUS is an effective and safe contraceptive method for nulliparous women, which also achieves high levels of satisfaction among patients. In addition, nulliparous women seem to experience fewer expulsions than parous ones. Bleeding pattern is acceptable for the majority of patients, and any bleeding profile disorders mainly occur in the first months after the insertion. Further in-depth, long-term prospective studies on different types of LNG-IUS in size and dosage are necessary in this patient group, to determine risk factors for the occurrence of side effects and associated discontinuations, which should not, however, delay the wider use of the method in this group, given the number of advantages and relatively few disadvantages.

## Figures and Tables

**Table 1:** The inclusion and exclusion criteria.

	Inclusion Criteria	Exclusion Criteria
Study Status	Completed, Published	Unfinished, Unpublished
Study Type	RCT non-RCT cohort study case-control study case-control study	eviewsr case reports case series letters to the editor expert opinions conference papers
Language	English	Other than English
Year Published	≥1990	<1990
Insertion	In scheduled mode	As an emergency contraception or immediately after intervention/surgery f.e. abortion
Topic	LNG-IUS as a long-term contraceptive method	LNG-IUS as a treatment f.e. of heavy menstrual bleeding

Abbreviations: f.e.: for example; LNG-IUS: levonorgestrel-releasing intrauterine system; RCT: randomized controlled trial.

**Table 2:** Characteristics of the studies included in the systematic review.

Authors & Year	Type of the Study	Main Aim	Population of Nulliparous LNG-IUS Users	Type of LNG-IUS	Observation/ Follow-Up Time
Pakarinen et al. 1996 [41]	Randomized prospective	To compare the efficacy, safety and acceptability of LNG-IUS situated in the cervical canal or uterine cavity	*N* = 145 69 LNG-IUS intracervical 76 LNG-IUS intrauterine	20 µg/24 h	1 year
Suhonen et al. 2004 [45]	Randomized prospective	To compare the safety and acceptability of LNG-IUS and OCs in young nulliparous women	*N* = 94 Median age: 21; Range: 18–25	Mirena 20 µg/24 h; Total content: 52 mg Size: 32 × 32 mm	1 year
Wildemeersch et al. 2005 [51]	Open prospective noncomparative	To evaluate the ease of insertion, contraceptive performance and the safety of LNG-IUS Femilis	*N* = 92 Mean age: 29 Range: 16–50	Femilis Slim 20 µg/24 h Total content: 40 mg Size: 30 × 24 mm	1 year
Römer et al. 2009 [42]	Cross-sectional	To identify the characteristics and experiences of women using LNG-IUS	~ 694 8% of 8680-whole study group	Mirena 20 µg/24 h Total content: 52 mg Size: 32 × 32 mm	20% used IUS < 1 year, 16% 1–2 years; 15% 2–3 years; 14% 3–4 years; 13% 4–5 years; 18% > 5 years; 3% no data
Wildemeersch et al. 2009 UPDATE ON: Wildemeersch et al. 2005 [50]	Open prospectivenoncomparative	To provide an update on the performance of LNG-IUS Femilis in parous and nulliparous women	*N* = 112 Mean age: 29 Range: 17–48	Femilis20 µg/24 hTotal content: 60 mgSize: 30 × 28 mm	5 years
Bahamondes et al. 2011 [32]	Retrospectivecohort	To evaluate the ease of insertion and clinical performance of LNG-IUS in nulligravidas for up to 1 year after insertion	*N* = 158 Mean age: 30	Mirena 20 µg/24 h Total content: 52 mg Size: 32 × 32 mm	1 year
Marions et al. 2011 [39]	Non-interventional cohort	To gain knowledge about the insertion and the use of LNG-IUS in nulliparous women	*N* = 224Median age: 20	Mirena 20 µg/24 h Total content: 52 mg Size: 32 × 32 mm	Follow-ups: 1st 2–5 weeks (*N* = 137) 2nd 12–26 weeks (*N* = 197) 3rd 30–124 weeks (optional) (*N* = 134)
Armitage et al. 2013 [31]	Observational prospective	To follow 100 women attending for fitting LNG-IUS at a single urban general practice serving students	*N* = 97 Age range: 18–38	Mirena 20 µg/24 h Total content: 52 mg Size: 32 × 32 mm	1 year
Madden et al. 2014 [38]	Secondary analysis of Contraceptive CHOICE Project [53]	To investigate if young age and nulliparity were associated with the expulsion of LNG or Cu-IUS	*N* = 1690	20 µg/24 h	1 year
Savasi et al. 2014 [43]	Retrospective	To assess complication rates with the use of LNG-IUS in adolescents with developmental disabilities	*N* = 54	Mirena 20 µg/24 h Total content: 52 mg Size: 32 × 32 mm	Follow-up after the insertion of IUD ranged from 0 to 51 months (15 months on average)
Zhao et al. 2014 [52]	Prospective	To analyze experiences and the levels of satisfaction with Mirena among Chinese women	*N* = 77	Mirena 20 µg/24 h Total content: 52 mg Size: 32 × 32 mm	Follow ups: 1st 3–4 months 2nd 1 year
Abraham et al. 2015 [30]	Secondary analysis of Contraceptive CHOICE Project [53]	To assess the relationship among young age, nulliparity and the continuation of LARC methods	*N* = 1456	Not specified	Follow ups: 1st 1 year 2nd 2 years
Kaislasuo et al. 2015 [37]	Prospective cohort	To assess if small uterine cavity size was associated with bleeding problems or pain in nulligravid women using IUD	*N* = 111	Mirena 20 µg/24 h Total content: 52 mg Size: 32 × 32 mm	1 year
Gemzell-Danielsson et al. 2015 [35]	Randomized prospective	To evaluate if the outcomes of LNG-IUS were affected by parity, age or BMI	*N* = 1130 Age range: 18–35	49%-LNG-IUS 8 µg/24 h Total content: 13.5 mg 51%-LNG-IUS 13 µg/24 h Total content: 19.5 mg Both size: 28 × 30 mm	3 years
Eisenberg et al. 2015 [34]	Partially randomized prospective	To assess 3-year data on the efficacy and safety of 52 mg LNG-IUS	*N* = 1011 at enrollment Mean age: 25 Range: 16–45	Liletta 20 µg/24 h Total content: 52 mg Size: 32 × 32 mm	3 years
Mejia et al. 2016 [40]	Secondary analysis of Contraceptive CHOICE Project [53]	To evaluate the effect of baseline bleeding patterns on the rates of amenorrhea during the use of 52 mg LNG-IUS	*N* = 515	Total content: 52 mg	1 year
Hall et al. 2016 [36]	Observational	To follow college students who chose IUD and assess the insertion, use, continuation, and satisfaction	*N* = 88 Aged 18–30	Mirena 20 µg/24 h Total content: 52 mg Size: 32 × 32 mm	1 year
Wildemeersch et al. 2017 [49]	Observational prospective	To report on the contraceptive performance of LNG-IUS after 5 years of use	*N* = 117 Mean age: 27 Range: 15–47	Femilis 20 µg/24 h Total content: 60 mg Size: 30 × 28 mm	5 years (*N* = 114)
Darney et al. 2018 [33]	Prospective cohort	To evaluate amenorrhea rates and predictors during the 1st year of LNG-IUS use	*N* = 822	Liletta 20 µg/24 h Total content: 52 mg Size: 32 × 32 mm	1 year
Schreiber et al. 2018 [44]	Prospective	To evaluate bleeding patterns for Liletta using the WHO Belsey definitions	*N* = 982	Liletta 20 µg/24 h Total content: 52 mg Size: 32 × 32 mm	First 90 days (*N* = 982) Second 90 days (*N* = 949) Last 90 days of year 1 (*N* = 866) Last 90 days of year 2 (*N* = 711) Last 90 days of year 3 (*N* = 568)
Vaitsiakhovich et al. 2018 [48]	Cross design analysis of randomized and observational data	To evaluate the discontinuation rate of LNG-IUS in real-life setting	*N* = 50	Mirena 20 µg/24 h Total content: 52 mg Size: 32 × 32 mm	2 years
Teal et al. 2019 UPDATE ON: Eisenberg et al. 2015 [46]	Partially randomizedprospective	To re-assess the efficacy and safety of 52 mg LNG-IUS after 5 years of use (the continuation of Eisenberg et al. 2015)	*N* = 1011 Mean age: 25 Range: 16–45	Liletta 20 µg/24 h Total content: 52 mg Size: 32 × 32 mm	5 years (*N* = 986)
Teunissen et al. 2019 [47]	Retrospective cohort	To investigate differences in continuation rates between the contraceptive and therapeutic use of LNG-IUS	*N* = 379	Total content: 52 mg	Minimum 5 years

Abbreviations: BMI: body mass index; Cu-IUS: Copper-releasing intrauterine system; IUS: intrauterine system; LNG-IUS: levonorgestrel releasing intrauterine system; OC: oral contraceptives; WHO: World Health Organization; LARC: long-acting reversible contraception; IUD: intrauterine devices.

**Table 3:** Continuation rates and reasons for discontinuation.

		Discontinuation Due to				
Authors & Year	Continuation Rate	Expulsion	Pain	Unacceptable Bleeding	Pregnancy Wish/Personal reasons	Other
Pakarinen et al. 1996 [41]	86.9% at 1st year					
Suhonen et al. 2004 [45]	79.8% at 1st year	1.1%	6.4%	2.1%	4.2%	Hormonal: 4.2% Other medical: 2.1%
Wildemeersch et al. 2005 [51]	90.2% at 1st year	1.1%	2.2%	2.2%	3.2%	Other: 1.1%
Bahamondes et al. 2011 [32]	92.0% at 1st year	3.7%	1.5%		2.4%	Other medical: 0.8% Lost to follow-up: 1.6%
Marions et al. 2011 [39]	73.1% at 1st year	3.0%	11.9%	10.4%		Infection/cyst: 1.5%
Armitage et al. 2013 [31]	77.3% at 1st year	2.1%	4.1%	2.1%		Lost to follow up: 10.3% Others: 4.1%
Savasi et al. 2014 [43]	92.6%-different observation time	1.9%	1.9%	5.6%		
Zhao et al. 2014 [52]	90.0% at 1st year					
Abraham et al. 2015 [30]	Younger than 20: 81.0% at 1st year 67.0% at 2nd year 20–25 years old: 87.0% at 1st year 79.0% at 2nd year Older than 25: 87.0% at 1st year 77.0% at 2nd year					
Kaislasuo et al. 2015 [37]	73.0% at 1st year	2.7%			1.8%	Other: 6.3% Lost to follow-up: 16.2%
Gemzell-Danielsson et al. 2015 [35]	LNG-IUS 8: 78.8% at 1st year 54.3% at 2nd year LNG-IUS 13: 79.8% at 1st year 58.1% at 2nd year					
Hall et al. 2016 [36]	93.1% at 1st year	1.4%				Side effects: 4.2% Lack of benefit: 1.4%
Wildemeersch et al. 2017 [49]	53.8% at 5th year	0%	6.0%		29.9%	Other medical: 6.8% Lost to follow up: 2.6% Pregnancy: 0.9%
Vaitsiakhovich et al. 2018 [48]	In RCT: 74.0% at 1st year 70.0% at 2nd year					
Teunissen et al. 2019 [47]	76.8% at 1st year 63.6% at 2nd year 50.4% at 3rd year 37.5% at 4th year 11.1% at 5th year					

Abbreviations: LNG-IUS: levonorgestrel-releasing intrauterine system; RCT: randomized controlled trial.

**Table 4:** Satisfaction rates.

Authors & Year	Assessment Time	Satisfaction Rate	Other
Suhonen et al. 2004 [45]	1st year	90% ‘moderately’ to ‘very good’	88% would like to continue
Römer et al. 2009 [42]	Various	93% ‘rather satisfied’ (31%) to‘very satisfied’ (62%) at various times	86% would recommend to a friend; 87% would like to continue
Marions et al. 2011 [39]	12–16 weeks	76% ‘very satisfied’ or ‘satisfied’; 10% ‘neither satisfied or dissatisfied’; 5% ‘dissatisfied’ 9% data missing	
Zhao et al. 2014 [52]	3–4 months 1st year	92% ‘very satisfied’ or ‘rather satisfied’ 85% ‘very satisfied’ or ‘rather satisfied’	
Gemzell-Danielsson et al. 2015 [35]	3rd year	LNG-IUS 8-94% ‘very satisfied’ or ‘somewhat satisfied’ LNG-IUS 13-96% ‘very satisfied’ or ‘somewhat satisfied’	LNG-IUS 8 -73% would like to continue LNG-IUS 13-80% would like to continue

Abbreviations: LNG-IUS: levonorgestrel-releasing intrauterine system.

## Supplementary Materials

The following are available online at https://www.mdpi.com/2077-0383/9/7/2101/s1, Figure S1. PRISMA 2009 Flow Diagram, Table S1. The search methods for used databases, Table S2. Risk of bias assessment.
