# Supplementary material for: Levonorgestrel-Releasing Intrauterine System as a Contraceptive Method in Nulliparous Women: A Systematic Review
Source: J Clin Med. 2020 Jul 3;9(7):2101. doi: 10.3390/jcm9072101 (PMC7408997; doi:10.3390/jcm9072101)
Supplement: Supplementary file 1 [file jcm-09-02101-s001.pdf]

Supplementary Materials

Table S1. The search methods for used databases.

Database	Number of retrieved studies	Search strategy
**PubMed**	173	(Intrauterine Devices[Mesh] OR intrauterine devic* OR intrauterine system* OR intrauterine contracept* OR IUD OR IUS OR Mirena OR Kyleena OR Skyla OR Jaydess OR Liletta OR Levosert) AND (Levonorgestrel[Mesh] OR levonorgestrel* OR norgestrel*) AND (Parity[Mesh] OR nullipar*) AND ("1990/01/01"[PDAT] : "2020/03/27"[PDAT])
**Scopus**	429	(TITLE-ABS-KEY (intrauterine AND devic* OR intrauterine AND system* OR intrauterine AND contracept* OR iud OR ius OR mirena OR kyleena OR skyla OR jaydess OR liletta OR levosert) AND (levonorgestrel* OR norgestrel*) AND (nullipar*)) AND (LIMIT-TO (PUBYEAR,2020) OR LIMIT-TO (PUBYEAR,2019) OR LIMIT-TO (PUBYEAR,2018) OR LIMIT-TO (PUBYEAR,2017) OR LIMIT-TO (PUBYEAR,2016) OR LIMIT-TO (PUBYEAR,2015) OR LIMIT-TO (PUBYEAR,2014) OR LIMIT-TO (PUBYEAR,2013) OR LIMIT-TO (PUBYEAR,2012) OR LIMIT-TO (PUBYEAR,2011) OR LIMIT-TO (PUBYEAR,2010) OR LIMIT-TO (PUBYEAR,2009) OR LIMIT-TO (PUBYEAR,2008) OR LIMIT-TO (PUBYEAR,2007) OR LIMIT-TO (PUBYEAR,2006) OR LIMIT-TO (PUBYEAR,2005) OR LIMIT-TO (PUBYEAR,2004) OR LIMIT-TO (PUBYEAR,2003) OR LIMIT-TO (PUBYEAR,2002) OR LIMIT-TO (PUBYEAR,2001) OR LIMIT-TO (PUBYEAR,2000) OR LIMIT-TO (PUBYEAR,1999) OR LIMIT-TO (PUBYEAR,1998) OR LIMIT-TO (PUBYEAR,1997) OR LIMIT-TO (PUBYEAR,1996) OR LIMIT-TO (PUBYEAR,1995) OR LIMIT-TO (PUBYEAR,1994) OR LIMIT-TO (PUBYEAR,1993) OR LIMIT-TO (PUBYEAR,1992) OR LIMIT-TO (PUBYEAR,1991) OR LIMIT-TO (PUBYEAR,1990)) AND (LIMIT-TO (DOCTYPE,"ar"))
**Embase**	155	(intrauterine AND devic* OR (intrauterine AND system*) OR (intrauterine AND contracept*) OR 'iud'/exp OR iud OR ius OR 'mirena'/exp OR mirena OR 'kyleena'/exp OR kyleena OR 'skyla'/exp OR skyla OR 'jaydess'/exp OR jaydess OR 'liletta'/exp OR liletta OR 'levosert'/exp OR levosert) AND (levonorgestrel* OR norgestrel*) AND nullipar* AND (1991:py OR 1992:py OR 1993:py OR 1995:py OR 1996:py OR 1997:py OR 1998:py OR 1999:py OR 2001:py OR 2002:py OR 2003:py OR 2004:py OR 2005:py OR 2006:py OR 2007:py OR 2008:py OR 2009:py OR 2010:py OR 2011:py OR 2012:py OR 2013:py OR 2014:py OR 2015:py OR 2016:py OR 2017:py OR 2018:py OR 2019:py OR 2020:py) AND ('article'/it OR 'article in press'/it)
**The Cochrane Central Register of Controlled Trials (CENTRAL)**	59	#1 Intrauterine Devices[Mesh]<br>#2 intrauterine device OR intrauterine system OR intrauterine contraception OR intrauterine contraceptive OR intrauterine contraceptives OR IUD OR IUS OR Mirena OR Kyleena OR Skyla OR Jaydess OR Liletta OR Levosert<br>#3 Levonorgestrel[Mesh]<br>#4 levonorgestrel OR norgestrel<br>#5 Parity[Mesh]<br>#6 nullipar*<br>#7 (#1 OR #2) AND (#3 OR #4) AND (#5 OR #6)<br>Filters: Trials; Year first publish range: 1990-2020

Table S2. Risk of bias assessment.

Assessed feature	Pakarinen et al. 1996	Suhonen et al. 2004	Wildemeersch et al. 2005	Römer et al. 2009	Wildemeersch et al. 2009	Bahamondes et al. 2011	Marions et al. 2011	Armitage et al. 2013	Madden et al. 2014	Savasi et al. 2014	Zhao et al. 2014	Abraham et al. 2015	Kaislasuo et al. 2015	Gemzell-Danielsson	Eisenberg et al. 2015	Mejia et al. 2016	Hall et al. 2016	Wildemeersch et al. 2017	Darney et al. 2018	Schreiber et al. 2018	Vaitsiakhovich et al. 2018	Teal et al. 2019	Teunissen et al. 2019
Selection																							
1) <u>Representativeness of the cohort</u> a) truly representative for LNG-IUS nulliparous users (≥500, without special features) **or** somewhat representative (≥200, without special features) ★ b) selected group of users e.g. with specific comorbidities d) no description	-	-	-	★	-	-	★	-	★	-	-	★	-	★	★	★	-	-	★	★	-	★	★
2) <u>Selection of the control group</u> a) drawn from the same community as the LNG-IUS nulliparous users ★ b) drawn from a different source c) no description	★	★	★	★	★	★	N/A*	★	★	N/A*	★	★	★	★	★	★	★	★	★	★	★	★	★
3) <u>Ascertainment of the exposure</u> a) yes, detailed description (e.g. type of LNG-	-	★	★	★	★	★	★	★	-	★	★	-	★	★	★	-	★	★	★	★	★	★	-

IUS, total content, dimensions) with medical validation ★<br>b) yes, e.g. record linkage or based on self-reports<br>c) no description																							
4) Demonstration that the outcomes of interest were not present at start of study<br>a) yes ★<br>b) no	★	★	★	N/A**	★	★	★	★	★	★	★	★	★	★	★	★	★	★	★	★	★	★	★
Comparability																							
1) Comparability of LNG-IUS nulliparous women to controls on the basis of the design or analysis<br>a) study controls for age, parity, LNG-IUS type★<br>b) study controls for at least one additional factor out of: gravidity, body mass index, baseline bleeding pattern ★<br>c) not reported	-	★★	★	-	★	★★	N/A*	-	★	N/A*	-	★	★	★★	★	-	-	★	-	-	★	★	-
Outcome																							
1) Ascertainment of outcomes<br>a) independent assessment **or** record linkage **or** self-report***	★	★	★	★	★	★	★	★	★	★	★	★	★	★	★	★	★	★	★	★	★	★	★

★<br>b) no description																							
2) <u>Was follow-up long enough for outcomes to occur</u><br>a) yes, at least 1 year ★<br>b) no	★	★	★	★	★	★	★	★	★	★	★	★	★	★	★	★	★	★	★	★	★	★	★
3) <u>Adequacy of follow up of cohorts</u><br>a) complete follow up **or** subjects lost to follow up unlikely to introduce bias - small number lost - >70 % follow up/proper description provided ★<br>b) follow up rate <70 or no description of lost<br>d) no statement	★	★	★	N/A** 	★	★	-	★	★	★	★	★	★	★	★	★	★	★	★	★	★	★	★
Result	★★★<br>★★	★★★<br>★★★<br>★★	★★★<br>★★★<br>★	★★★<br>★★	★★★<br>★★★<br>★	★★★<br>★★★<br>★★	★★★<br>★★	★★★<br>★★★	★★★<br>★★★<br>★	★★★<br>★★	★★★<br>★★★	★★★<br>★★★<br>★	★★★<br>★★★<br>★	★★★<br>★★★<br>★★★	★★★<br>★★★<br>★★	★★★<br>★★★	★★★<br>★★★	★★★<br>★★★<br>★	★★★<br>★★★<br>★	★★★<br>★★★<br>★	★★<br>★<br>★★<br>★<br>★	★★<br>★<br>★★<br>★<br>★★	★★<br>★<br>★★<br>★
Maximum number of stars	★★★<br>★★★<br>★★★	★★★<br>★★★<br>★★★	★★★<br>★★★<br>★★★	★★★<br>★★★<br>★	★★★<br>★★★<br>★★★	★★★<br>★★★<br>★★★	★★★<br>★★★	★★★<br>★★★<br>★★★	★★★<br>★★★<br>★★★	★★★<br>★★★	★★★<br>★★★<br>★★★	★★★<br>★★★<br>★★★	★★★<br>★★★<br>★★★	★★★<br>★★★<br>★★★	★★★<br>★★★<br>★★★	★★★<br>★★★<br>★★★	★★★<br>★★★<br>★★★	★★★<br>★★★<br>★★★	★★★<br>★★★<br>★★★	★★★<br>★★★<br>★★★	★★<br>★<br>★★<br>★<br>★★<br>★	★★<br>★<br>★★<br>★<br>★★<br>★	★★<br>★<br>★★<br>★<br>★★<br>★
Risk of bias for the interpretation of results for nulliparous	high	low	low	high	low	low	moderate	high	low	moderate	high	low	low	low	low	high	high	low	high	high	low	low	high

Note: This is a modified Newcastle - Ottawa Quality Assessment Scale [1]. It has been modified to assess the risk of bias in studies assessing performance of levonorgestrel-releasing intrauterine system as a contraceptive method in nulliparous women. Note: A study can be awarded one star for each numbered item within the Selection and Outcome categories. A maximum of two stars can be awarded for Comparability.

1.GA Wells, B.S., D O'Connell, J Peterson, V Welch, M Losos, P Tugwell,. The Newcastle-Ottawa Scale (NOS) for assessing the quality of nonrandomised studies in meta-analyses. *http://www.ohri.ca/programs/clinical_epidemiology/oxford.asp* **s**tate for the 20th April 2020.

*only the group of interest included in the study. **one point assessment. ***star was assigned if the self-report concerned subjective feelings, e.g. the level of satisfactio.n

Supplementary File 2.

PRISMA 2009 Flow Diagram

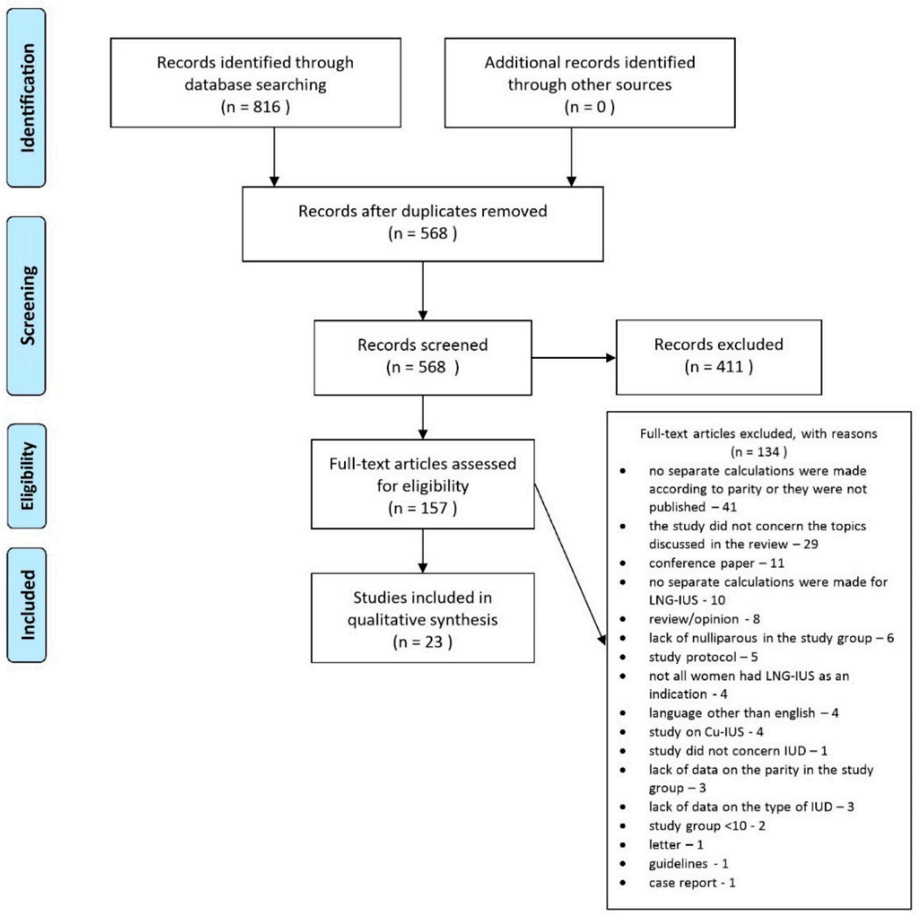

From: Moher D, Liberati A, Tetzlaff J, Altman DG, The PRISMA Group (2009). *Preferred Reporting Items for Systematic Reviews and Meta-Analyses*: The PRISMA Statement. PLoS Med 6(7): e1000097. doi:10.1371/journal.pmed1000097

For more information, visit www.prisma-statement.org.

Figure S1. PRISMA 2009 Flow Diagram.
